# Connectivity of the Cerebello-Thalamo-Cortical Pathway in Survivors of Childhood Leukemia Treated With Chemotherapy Only

**DOI:** 10.1001/jamanetworkopen.2020.25839

**Published:** 2020-11-20

**Authors:** Nicholas S. Phillips, Shelli R. Kesler, Matthew A. Scoggins, John O. Glass, Yin Ting Cheung, Wei Liu, Pia Banerjee, Robert J. Ogg, Deokumar Srivastava, Ching-Hon Pui, Leslie L. Robison, Wilburn E. Reddick, Melissa M. Hudson, Kevin R. Krull

**Affiliations:** 1 Department of Epidemiology and Cancer Control, St Jude Children’s Research Hospital, Memphis, Tennessee; 2 Department of Oncology, St Jude Children’s Research Hospital, Memphis, Tennessee; 3 Now with School of Nursing, University of Texas at Austin; 4 Department of Neuro-oncology, University of Texas MD Anderson Cancer Center, Houston; 5 Department of Diagnostic Imaging, St Jude Children’s Research Hospital, Memphis, Tennessee; 6 School of Pharmacy, Faculty of Medicine, Chinese University of Hong Kong, Hong Kong, China; 7 Department of Biostatistics, St Jude Children’s Research Hospital, Memphis, Tennessee; 8 Department of Psychology, St Jude Children’s Research Hospital, Memphis, Tennessee

## Abstract

**Importance:**

Treatment with contemporary chemotherapy-only protocols is associated with risk for neurocognitive impairment among survivors of childhood acute lymphoblastic leukemia (ALL).

**Objective:**

To determine whether concurrent use of methotrexate and glucocorticoids is associated with interference with the antioxidant system of the brain and damage and disruption of glucocorticoid-sensitive regions of the cerebello-thalamo-cortical network.

**Design, Setting, and Participants:**

This cross-sectional study was conducted from December 2016 to July 2019 in a single pediatric cancer tertiary care center. Participants included survivors of childhood ALL who were more than 5 years from cancer diagnosis, age 8 years or older, and treated on an institutional chemotherapy-only protocol. Age-matched community members were recruited as a control group. Data were analyzed from August 2017 to August 2020.

**Exposure:**

ALL treatment using chemotherapy-only protocols.

**Main Outcomes and Measures:**

This study compared brain volumes between survivors and individuals in a community control group and examined associations among survivors of methotrexate and dexamethasone exposure with neurocognitive outcomes. Functional and effective connectivity measures were compared between survivors with and without cognitive impairment. The Rey-Osterrieth complex figure test, a neurocognitive evaluation in which individuals are asked to copy a figure and then draw the figure from memory, was scored according to published guidelines and transformed into age-adjusted *z* scores based on nationally representative reference data and used to measure organization and planning deficits. β values for neurocognitive tests represented the amount of change in cerebellar volume or chemotherapy exposure associated with 1 SD change in neurocognitive outcome by *z* score (mm^3^/1 SD in *z* score for cerebellum, mm^3^/[g×hr/L] for dexamethasone and methotrexate AUC, and mm^3^/intrathecal count for total intrathecal count).

**Results:**

Among 302 eligible individuals, 218 (72%) participated in the study and 176 (58%) had usable magnetic resonance imaging (MRI) results. Among these, 89 (51%) were female participants and the mean (range) age was 6.8 (1-18) years at diagnosis and 14.5 (8-27) years at evaluation. Of 100 community individuals recruited as the control group, 82 had usable MRI results; among these, 35 (43%) were female individuals and the mean (range) age was 13.8 (8-26) years at evaluation. There was no significant difference in total brain volume between survivors and individuals in the control group. Survivors of both sexes showed decreased mean (SD) cerebellar volumes compared with the control population (female: 70 568 [6465] mm^3^ vs 75 134 [6780] mm^3^; *P* < .001; male: 77 335 [6210] mm^3^ vs 79 020 [7420] mm^3^; *P* < .001). In female survivors, decreased cerebellar volume was associated with worse performance in Rey-Osterrieth complex figure test (left cerebellum: β = 55.54; SE = 25.55; *P* = .03; right cerebellum: β = 52.57; SE = 25.50; *P* = .04) and poorer dominant-hand motor processing speed (ie, grooved pegboard performance) (left cerebellum: β = 82.71; SE = 31.04; *P* = .009; right cerebellum: β = 91.06; SE = 30.72; *P* = .004). In female survivors, increased number of intrathecal treatments (ie, number of separate injections) was also associated with Worse Rey-Osterrieth test performance (β = −0.154; SE = 0.063; *P* = .02), as was increased dexamethasone exposure (β = −0.0014; SE = 0.0005; *P* = .01). Executive dysfunction was correlated with increased global efficiency between smaller brain regions (Pearson *r* = −0.24; *P* = .01) compared with individuals without dysfunction. Anatomical connectivity showed differences between impaired and nonimpaired survivors. Analysis of variance of effective-connectivity weights identified a significant interaction association (*F* = 3.99; *P* = .02) among the direction and strength of connectivity between the cerebellum and DLPFC, female sex, and executive dysfunction. Finally, no effective connectivity was found between the precuneus and DLPFC in female survivors with executive dysfunction.

**Conclusions and Relevance:**

These findings suggest that dexamethasone exposure was associated with smaller cerebello-thalamo-cortical regions in survivors of ALL and that disruption of effective connectivity was associated with impairment of executive function in female survivors.

## Key Points

**Question:**

Are changes in glucocorticoid receptor–rich brain structures of the cerebello-thalamo-cortical network associated with altered network communication and neurocognitive performance in survivors of childhood acute lymphoblastic leukemia (ALL) treated using chemotherapy-only protocols?

**Findings:**

In this cross-sectional study of 176 survivors of childhood ALL treated with chemotherapy only, a significant interaction association was found among female survivors between glucocorticoid exposure and altered functional and effective connectivity of the cerebellum and dorsolateral frontal cortex.

**Meaning:**

These findings suggest that female survivors of ALL may be at increased risk of altered connectivity from glucocorticoid exposure during therapy and may benefit from posttreatment strategies that repair altered network communication in the cerebello-thalamo-cortical network.

## Teaser

This cross-sectional study examines associations among brain volume, brain network connectivity, and neurocognitive test performance in survivors of childhood acute lymphoblastic leukemia treated with chemotherapy only.

## Introduction

Among survivors of childhood acute lymphoblastic leukemia (ALL), treatment with contemporary chemotherapy-only protocols, which include high doses of methotrexate and glucocorticoids (ie, prednisone, hydrocortisone, and dexamethasone), is associated with increased risk for neurocognitive impairment.^1^ Previously, we examined supratentorial connectivity in survivors of ALL treated with chemotherapy only and found that poor network segregation and specialization of brain regions were associated with increased numbers of triple-therapy (ie, methotrexate, glucocorticoid, and cytarabine) intrathecal administrations and executive dysfunction.^2^ Additionally, among survivors of childhood ALL, female sex has been associated with higher frequency of executive dysfunction.^3^ The pathophysiology underlying this association remains unclear.

Concomitant use of glucocorticoids and methotrexate is associated with blocking or depleting key enzymes in the glutathione antioxidant pathway via direct or indirect action. Glucocorticoids are associated with decreased activity of glutathione peroxidase in the antioxidant pathway, which is associated with decreased cellular ability to clear oxidants.^4,5^ Glucocorticoids are indirectly associated with increased sensitivity of brain cells to glutamate toxicity via presynaptic glutamate release.^6,7^ Excess glutamate is associated with inhibition among cystine/glutamate transporters, which is associated with decreased levels of glutathione and accumulation of lipid peroxides and reactive oxygen species.^8^ Methotrexate is associated with decreased glucose-6-phosphate dehydrogenase activity in the pentose phosphate cycle and glutathione reductase activity, which is important for preventing intracellular accumulation of peroxide.^9^ This double hit, in glucocorticoid receptor–rich brain regions, may be associated with antioxidant/oxidant imbalance and accumulation of peroxide. Consistent with our model, we previously reported the association of increased cerebrospinal fluid levels of oxidized phosphatidylcholine (a marker associated with oxidative stress) with induction treatment and consolidation among survivors of ALL treated with chemotherapy only.^10^ These increased levels were associated with neurocognitive deficits in working memory, organization, and attention.^11^ We suggested that neural networks rich in glucocorticoid receptors may be particularly at risk of damage from methotrexate and glucocorticoid exposure in survivors of ALL.

The distribution of glucocorticoid receptors in the brain is not homogenous.^12^ The hippocampus and cerebellum have the highest concentrations of glucocorticoid receptors. We have previously demonstrated^13^ that survivors of childhood ALL treated on chemotherapy-only protocols had smaller hippocampal, cerebellar, anterior cingulate, precuneus, and dorsolateral prefrontal cortex (DLPFC) regions compared with age-matched healthy individuals in control groups. This finding is consistent with studies that showed that glucocorticoid exposure is associated with smaller brain structures and attenuated functional activity in populations without cancer.^14,15,16,17^ Additionally, studies have shown that these structures are associated with each other structurally and functionally.^18,19,20,21^

Increasing evidence suggests that the cerebellum may regulate activity and facilitate effective neural communication by synchronizing functionally associated supratentorial neural clusters in the cerebral cortex.^22,23,24^ These networks organize to facilitate economical signaling, and they serve specific cognitive functions.^25,26^ Alterations to these networks are associated with inefficient information processing and decreased neurocognitive performance.^27^ Evidence suggests that the frontoparietal network has increased associations with cerebellar integrity compared with other neural networks.^22,23^ Treatment-induced injury to these cerebello-thalamo-cortical networks could therefore be associated with disruption of synchronized signaling and neurocognitive impairment.^28^ Evidence for the role of the cerebello-thalamo-cortical network in neurocognitive function has been described by Schmahmann and Sherman,^29^ who found that cerebellar damage was associated with deficits in a constellation of cognitive functions, including working memory, cognitive flexibility, visuospatial integration, language, and global intelligence.

In this study, we performed a targeted, hypothesis-driven network analysis to investigate associations among functional and effective connectivity, treatment exposures, and neurocognitive function in survivors of childhood ALL. We previously found that being a survivor of ALL was associated with decreased size in regions of the cerebello-thalamo-cortical network compared with age-matched individuals who did not have ALL.^13^ We hypothesized that decreased size in these regions would be associated with neurocognitive impairment and would vary by treatment intensity. Furthermore, we hypothesized that the cerebellum facilitates neural communication (ie, effective connectivity) between the DLPFC and the precuneus and that this would be associated with executive dysfunction in survivors of ALL.

## Methods

### Study Design and Participants

This cross-sectional study protocol was approved by the institutional review board of St Jude Children’s Research Hospital, and all participants provided written informed consent or assent with informed consent from the parent or guardian. The study follows the Strengthening the Reporting of Observational Studies in Epidemiology (STROBE) reporting guideline.

In this study, conducted from December 2016 to July 2019, participants were recruited among survivors of ALL treated in the St Jude Children’s Research Hospital Total Therapy Study XV (TOTXV) protocol.^30^ Survivors were considered eligible if they had received a cancer diagnosis 5 or more years previously and were aged 8 years or older. The TOTXV protocol was a risk-stratified, institution-based, chemotherapy-only protocol that omitted cranial radiation therapy in all patients.^31^ Patients in this protocol were treated with oral dexamethasone and intrathecal glucocorticoids and methotrexate. This study evaluated intravenous methotrexate, oral dexamethasone, and intrathecal glucocorticoids and methotrexate. Exclusion criteria included death prior to long-term follow-up, secondary cancers or relapse requiring cranial radiation or additional chemotherapy, unrelated central nervous system injury or disease, lack of proficiency in English, and loss of eligibility for pediatric follow-up. Age- and sex-matched individuals who were aged 6 to 25 years were recruited from the local community for a control population and were excluded if not proficient in English or if diagnosed with central nervous system injury or disease. Individuals in the control population were matched to the study patient population by socioeconomic status (SES) using the Barratt simplified measure of social status.

### Pharmacological Analysis

Blood samples were drawn at 0, 6, 23, and 42 hours surrounding each administration of intravenous high-dose methotrexate infusion. Blood samples for dexamethasone were collected prior to oral administration and at 1, 2, 4, and 8 hours after the morning dose on days 1 and 8 of reinduction I in weeks 7 and 8 of continuous therapy.^32^ Dexamethasone and methotrexate levels were measured in serum and quantified as area under curve (AUC), as previously described.^33,34^

### Imaging

Survivors were assessed once during long-term follow-up, from 5 to 10 years after diagnosis. Survivors and individuals in the control population were imaged using structural magnetic resonance imaging (MRI), including a T1-weighted set of imaging results acquired using sagittal 3-dimensional magnetization-prepared rapid acquisition with gradient echo (MPRAGE) sequence (repetition time [TR]/echo time [TE]/inversion time = 1980/2.32/1100 ms), with an imaging resolution of 1.0 mm isotropic. In survivors, resting-state functional MRI (fMRI) was obtained during 6 minutes of eyes-open rest on a 3T scanner (Siemens Trio or Skyra) using a single-shot T2*-weighted echo-planar pulse sequence (TR, 2.06 seconds; TE, 30 milliseconds; field of view, 192 mm; matrix, 64 × 64; slice thickness, 4 mm). Resting-state images were processed using statistical parametric mapping with SPM statistical software version 8 (Wellcome Centre for Human Neuroimaging), as previously described (eAppendix in the Supplement).^27,35,36,37^

### Neurocognitive Testing

Standardized neuropsychological testing was conducted within 1 day of brain imaging in survivors and the control population. A certified psychological examiner conducted testing under supervision of a board-certified neuropsychologist (K.R.K). As part of a larger battery of tests, the examiner administered the Delis-Kaplan Executive Function System^38^ and processing-speed and working-memory subtests from Wechsler Intelligence Scale for Children Fourth Edition and Wechsler Adult Intelligence Scale Fourth Edition.^39^ General results from these tests were reported in a 2016 study.^40^ Raw scores were transformed into age-corrected standard scores based on population reference values. Dominant hand motor processing speed was evaluated using grooved pegboard test performance, a neurocognitive evaluation of eye-hand coordination and motor speed in which individuals are asked to insert grooved pegs into matching grooved holes as quickly as possible, scored according to published guidelines and transformed into age-adjusted *z* scores based on nationally representative reference data. Executive dysfunction was defined as an age-adjusted *z* score below the 10th percentile of the nationally representative *z* score for that age group in letter/number switch, color/word switch, verbal fluency, digit span backward, Rey-Osterrieth complex figure, or 20 questions measures. In the Rey-Osterrieth complex figure test, individuals are asked to copy a complex figure and then draw it from memory immediately and again after a small delay. It was scored according to published guidelines and transformed into age-adjusted *z* scores based on nationally representative reference data; it was used to measure organization and planning deficits. Neurocognitive outcomes are presented by test measurement and grouped, for ease of comparison, by the theoretical construct used in a 2016 study.^32^

### Functional Connectivity Analysis

Our aim was to evaluate specific a priori functional subnetworks (ie, modules) within a brain network (ie, connectome). Accordingly, we defined subnetworks of the cerebello-thalamo-cortical network as bilateral cerebellar Crus I and II, bilateral thalamus, bilateral DLPFC, and bilateral precuneus using the automated anatomical atlas.^41^ To reduce confounders and improve power, we compared network connectivity in survivors who had executive dysfunction with that of individuals without executive dysfunction. We also examined a language network (consisting of bilateral Brodmann areas 40, 44, and 45) as a control comparison, given that this network has relatively low glucocorticoid receptor distribution and survivors demonstrate few if any expressive or receptive language problems. Functional connectivity matrices for the regions of interest were obtained using Conn toolbox version 18.4 (Alfonso Nieto-Castanon), as previously described.^22,36,37^ Global efficiency of the bilateral cerebello-thalamo-cortical network was calculated using a permutation distribution, with the *P* value calculated by determining the proportion of times the permutation mean difference was greater than the actual mean difference (out of 2000 permutations); permutation mean differences outside the confidence interval were considered significant.

### Effective Connectivity Analysis

To test the hypothesis that cerebellar activity is associated with connectivity between the DLPFC and the precuneus, we performed an analysis of effective connectivity. We used bayesian network analysis to identify directional and weighted network structure among the regions of interest (ie, cerebellum, precuneus, and DLPFC). This technique has been used to identify biologically relevant structure-function dependencies.^42,43^ Time courses were extracted from a 50-element independent-component analysis. Effective connectivity was estimated using a bayesian network score–based structure-learning algorithm for survivors with impairment (ie, score on any executive function test less than −1.3) and survivors without impairment, stratified by sex, to identify differences in network structure across subgroups. Additionally, bayesian network analysis was performed on the entire cohort to find the best-fit common model and to estimate connectivity weights for subsequent 2-way analysis of variance (ANOVA) comparison (by sex and by impaired vs not impaired) (eAppendix in the Supplement).

### Statistical Analysis

General linear models were used to compare demographic characteristics and brain volumes between survivors and members of the control group. Among survivors, sex-stratified multivariable linear models were used to test associations among morphometric measurements and serum concentration of dexamethasone and methotrexate AUC, adjusting for age at diagnosis, age at assessment, and intracranial volume. Regions of interest were identified a priori based on glucocorticoid receptor distribution and inclusion in the cerebello-thalamo-cortical pathway. A multivariable generalized linear model was used to examine associations between morphometric measurements and neurocognitive scores. β values for neurocognitive tests represent the amount of change in cerebellar volume or chemotherapy exposure associated with 1 SD change in neurocognitive outcome by z score (mm^3^/1 SD in *z* score for cerebellum, mm^3^/[g×hr/L] for dexamethasone and methotrexate AUC, and mm3/intrathecal count for total intrathecal count). Statistical models included age at diagnosis, age at assessment, intracranial volume, dexamethasone serum levels, methotrexate serum levels, and number of intrathecal injections received during therapy as covariates. Correlation coefficients were calculated between global efficiency metrics for treatment and neurocognitive outcomes. All results were corrected for false discovery rate by Benjamini, Hochberg, and Yekutieli method and closed testing procedures as applicable. Statistical analysis was conducted from August 2017 to August 2020 using RStudio Team 2017, including RStudio statistical software version 1.1 (RStudio PBC). Two-sided *t* tests and 1-sided generalized linear modeling were conducted with a statistical significance level of *P* < .05.

## Results

### Demographic and Anatomic Differences

Of 408 survivors treated at St. Jude on the TOTXV protocol, 302 individuals (74.0%) were eligible for this study, and of 218 individuals who participated (72.2%), 38 individuals (17.4%) had MRIs with artifacts or incomplete images and 4 individuals had incomplete segmentation. The remaining 176 survivors of ALL included in this study had a mean (range) age of 6.8 (1-18) years at diagnosis and 14.5 (8-27) years at evaluation; 89 [51%] were female individuals. These survivors of ALL were compared with 82 age- and SES-matched community members as a control population. The mean (range) age of the control population was 13.8 (8-26) years at evaluation, and 35 [43%] were female individuals.(Table 1). There were no significant differences in demographic characteristics between groups. Mean SES for the control population was within 10% of patient SES. Survivors were a mean (SD) of 7.7 (1.7) years from diagnosis. No difference was found in intracranial volume between survivors and individuals in the control group, suggesting comparable global brain volumes. Mean (SD) cerebellar volume in survivors, compared with the age-matched control population, was significantly smaller in both sexes (male: 77 334 [6210] mm^3^ vs 79 019 [7420] mm^3^; *P* < .001; female: 70 568 [6465] mm^3^ vs. 75 134 [6780] mm^3^; *P* < .001). 

**Table 1.** Characteristics and Comparison of Anatomic Locations Associated With the Cerebello-Thalamo-Cortical Network Between Survivors and Control Population

Characteristic	Mean (SD)		*P* value
	Survivors (n = 176)	Control population (n = 82)	
**Demographic characteristic**			
Sex, No. (%)			
Male	87 (49.4)	47 (57.3)	.47
Female	89 (50.6)	35 (42.7)	.86
Race/Ethnicity, No. (%)			
White	162 (92.0)	73 (89.0)	.43
Black or other	14 (8.0)	9 (11.0)	
Age, y			
At diagnosis	6.8 (4.5)	NA	NA
At evaluation	14.5 (4.8)	13.8 (4.8)	.85
Time since diagnosis, y	7.7 (1.7)	NA	NA
**Anatomic location**			
Female			
Cerebellum volume, mm^3^			
Left	70 611 (6540)	75 083 (6030)	<.001
Right	70 525 (6390)	75 186 (7530)	<.001
Thalamus volume, mm^3^			
Left	7457 (801)	7658 (870)	.11
Right	7546 (971)	7812 (805)	.07
Precuneus thickness, mm			
Left	2.48 (0.35)	2.65 (0.22)	<.001
Right	2.59 (0.17)	2.59 (0.17)	<.001
DLPFC thickness, mm			
Left	2.88 (0.19)	2.96 (0.18)	.014
Right	2.88 (0.20)	2.98 (0.17)	.003
Male			
Cerebellum volume, mm^3^			
Left	77 437 (6030)	80 602 (7380)	.003
Right	77 232 (6390)	77 437 (7460)	.002
Thalamus volume, mm^3^			
Left	8001 (829)	8171 (1002)	.14
Right	8142 (907)	8344 (917)	.10
Precuneus thickness, mm			
Left	2.60 (0.18)	2.73 (0.20)	<.001
Right	2.57 (0.17)	2.71 (0.17)	<.001
DLPFC thickness, mm			
Left	2.86 (0.17)	3.00 (0.19)	<.001
Right	2.85 (0.16)	2.98 (0.21)	<.001

Abbreviations: DLPFC, dorsolateral prefrontal cortex; NA, not applicable.

### Neurocognitive Outcomes

Executive function scores and processing speed were significantly decreased for survivors compared with population reference values (Table 2). For example, scores for survivors in the executive function test for verbal fluency were significantly decreased compared with expected population values (male survivors: mean [SD] age-adjusted *z* score, −0.49 [1.03]; *P* < .001; female survivors: mean [SD] age-adjusted *z* score, −0.25 [0.93]; *P* = .04). Scores for survivors in the dominant-hand processing-speed test were significantly decreased compared with expected population values (male survivors: mean [SD] age-adjusted *z* score, −1.48 [1.50]; *P* < .001; female survivors: mean [SD] age-adjusted *z* score, −1.16 [1.57]; *P* < .001). Additionally, scores for survivors in the Rey-Osterrieth complex figure test were significantly decreased compared with expected population values (male survivors: mean [SD] age-adjusted *z* score, −2.44 [2.37]; *P* < .001; female survivors: mean [SD] age-adjusted *z* score, −2.33 [2.45]; *P* < .001). Among female survivors, decreased bilateral cerebellar volume was associated with poorer Rey-Osterrieth complex figure performance (left cerebellum: β = 55.54; SE = 25.55; *P* = .03; right cerebellum: β = 52.57; SE = 25.50; *P* = .04), as was increased dexamethasone exposure (β = −0.0014; SE = 0.0005; *P* = .01) and increased number of triple intrathecal chemotherapy treatments (β = −0.154; SE = 0.063; *P* = .02). For female survivors, decreased bilateral cerebellar volume was associated with poorer dominant-hand motor-processing speed (ie, grooved pegboard performance) (left cerebellum: β = 82.71; SE = 31.04; *P* = .009; right cerebellum: β = 91.06; SE = 30.72; *P* = .004) (Table 3). Among male survivors, decreased bilateral cerebellar volumes were associated with poorer symbol search processing speed (left cerebellum: β = 49.57; SE = 18.06; *P* = .007; right cerebellum: β = 47.15; SE = 18.31; *P* = .01), decreased right cerebellar volume was associated with poorer digit backward performance (β = 35.04; SE = 17.06; *P* = .04), and decreased left cerebellar volume was associated with poorer dominant-hand motor-processing speed (β = 56.98; SE = 26.88; *P* = .04). In male survivors, increased triple intrathecal treatments was associated with poor dominant-hand motor speed (β = −0.059; SE = 0.044; *P* = .02).

**Table 2.** Neurocognitive Outcomes of Survivors of Childhood Acute Lymphoblastic Leukemia After Completion of Therapy Compared With National Reference Values

Outcome	Male (n = 108)			Female (n = 104)				
	Mean (SD)^a^	*P* value^b^^,^^c^	Impairment (95% CI)^b^^,^^d^		Mean (SD)^a^	*P* value^b^^,^^c^	Impairment (95% CI)^b^^,^^d^	Male vs female *P* value^b^^,^^c^
**Executive function**								
Flexibility								
Number-letter switch	−0.74 (1.14)	<.001	30.48 (21.67-39.28)	−0.38 (1.23)		.02	24.04 (15.83 to 32.25)	.13
Color-word switch	−0.34 (0.95)	.002	14.85 (7.92-21.79)	−0.07 (1.03)		.68	13.59 (6.97-20.21)	.19
Fluency								
Verbal	−0.49 (1.03)	<.001	31.73 (22.79-40.68)	−0.25 (0.93)		.04	16.35 (9.24-23.45)	.21
Categorical	0.03 (1.15)	.78	16.35 (9.24-23.45)	−0.01 (0.99)		.92	12.5 (6.14-18.86)	.84
Working memory								
Digit backward	−0.33 (1.03)	.004	20 (12.35-27.65)	−0.25 (0.96)		.04	14.42 (7.67-21.18)	.80
Spatial backward	−0.05 (0.98)	.64	10.48 (4.62-16.33)	−0.02 (1.00)		.86	14.42 7.67-21.18)	.85
Organization and planning								
Rey-Osterrieth complex figure copy	−2.44 (2.37)	<.001	62.86 (53.62-72.10)	−2.33 (2.45)		<.001	54.37 (44.75-63.99)	.84
20 Questions	−0.12 (1.11)	.31	19.23 (11.66-26.81)	−0.24 (0.94)		.04	16.5 (9.34-23.67)	.68
Tower	−0.18 (0.87)	.06	13.21 (6.76-19.65)	−0.05 (0.83)		.68	7.69 (2.57-12.81)	.49
**Processing speed**								
Motor								
Dominant hand	−1.48 (1.50)	<.001	48.57 (39.01-58.13)	−1.16 (1.57)		<.001	35.92 (26.66-45.19)	.28
Visual								
Symbol search	−0.25 (1.06)	.029	17.92 (10.62-25.23)	0.14 (0.99)		.27	8.65 (3.25-14.06)	.04
Visual-motor								
Digit symbol	−0.70 (0.90)	<.001	25.71 (17.35-34.07)	−0.10 (0.93)		.42	8.65 (3.25-14.06)	<.001
Number sequencing	−0.25 (0.99)	.02	19.05 (11.54-26.56)	−0.18 (1.15)		.22	17.31 (10.04-24.58)	.80
Letter sequencing	−0.47 (1.18)	<.001	24.76 (16.51-33.02)	−0.23 (1.12)		.09	16.35 (9.24-23.45)	.28

^a^ Age-adjusted *z* scores referenced to nationally representative reference values.

^b^ Corrected for false-discovery rate.

^c^ Comparison of group with expected population value (μ = 0; σ = 1.0) for test.

^d^ A score below the 10th percentile of the age-adjusted *z* score.

**Table 3.** Clinically Significant Neurocognitive Deficits in Survivors Associated With Decreased Cerebellar Volume and Chemotherapy, Controlling for Intracranial Volume and Age at Diagnosis

Outcome	Cerebellum				Dexamethasone AUC		Total intrathecal count		Methotrexate AUC	
	Left		Right							
	β (SE)^a^	*P* value	β (SE)^a^	*P* value	β (SE)^b^	*P* value	β (SE)^c^	*P* value	β (SE)^b^	*P* value
**Digit backward**										
Male	34.40 (17.45)	.05	35.04 (17.06)	.04	0.0003 (0.0005)	.53	0.020 (0.030)	.50	0.0012 (0.0093)	.90
Female	7.80 (18.51)	.68	7.22 (18.50)	.70	−0.0005 (0.0004)	.18	−0.006 (0.045)	.90	0.0087 (0.0113)	.44
**Rey-Osterrieth complex figure**										
Male	2.57 (38.35)	.95	7.05 (37.58)	.85	0.0010 (0.0011)	.36	0.005 (0.040)	.90	−0.0029 (0.0202)	.89
Female	55.54 (25.55)	.03	52.57 (25.50)	.04	−0.0014 (0.0005)	.01	−0.154 (0.063)	.02	−0.0009 (0.0304)	.98
**Dominant hand motor speed**										
Male	56.98 (26.88)	.04	50.92 (25.53)	.06	0.0011 (0.0008)	.15	−0.059 (0.044)	.02	−0.0053 (0.0135)	.69
Female	82.71 (31.04)	.009	91.06 (30.72)	.004	−0.0001 (0.0006)	.83	−0.090 (0.070)	.20	0.0242 (0.0173)	.17
**Symbol search**										
Male	49.57 (18.06)	.007	47.15 (18.31)	.01	0.0004 (0.0005)	.42	−0.054 (0.026)	.06	0.0019 (0.0094)	.84
Female	4.90 (19.66)	.80	1.13 (19.66)	.95	−0.0007 (0.0004)	.08	−0.042 (0.045)	.35	−0.0027 (0.0117)	.82

Abbreviation: AUC, area under the curve.

^a^ mm^3^/1-SD in *z* score.

^b^ mm^3^/(g×hr/L).

^c^ mm^3^/intrathecal count.

### Functional Connectivity Analysis

Of 176 survivors, 23 individuals (13%) (including 14 female and 9 male survivors) showed completely disconnected nodes in the left and right hemisphere cerebello-thalamo-cortical networks, even at the lowest-density threshold required to remove false-positive connections. Among 23 survivors with completely disconnected nodes, mean (range) age at diagnosis of ALL was 5.4 (2.1-9.2) years for female survivors and 9.6 (3.9-18.2) years for male survivors. Female survivors with disconnected nodes had decreased right DLPFC thickness compared with individuals in the control population (mean difference, −0.336; *P* = .045). Male survivors with disconnected nodes had decreased right cerebellar volume compared with individuals in the control population, as measured by volume ratio controlling for intracranial volume (0.044 vs 0.0294; *P* = .03). This fragmentation was not significantly different in survivors with executive dysfunction compared with those without executive dysfunction.

**Figure 1.**
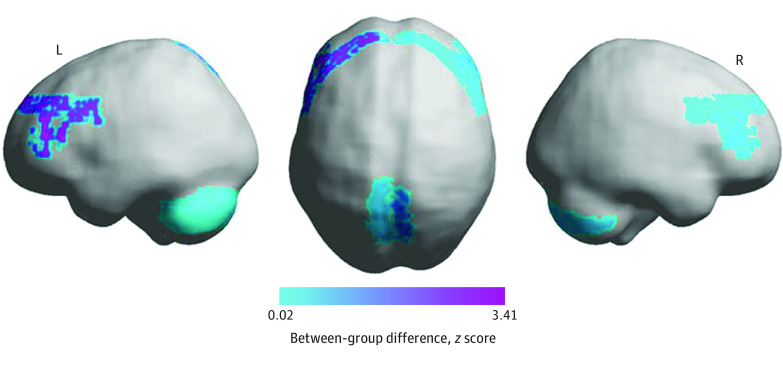
Surface Image of Cerebello-Thalamo-Cortical Functional Connectivity Differences in Survivors Purple shades indicate greater between-group difference of individuals with executive function impairment compared with those without executive function impairment. Areas in the left dorsolateral prefrontal cortex show increased differences in survivors with impairment compared with survivors without impairment. Image shown in neurologic convention. L indicates, left; R, right.

Among all study survivors, mean (SD) global efficiency of the bilateral cerebello-thalamo-cortical network was increased compared with a control network that had lower glucocorticoid receptor distribution (ie, Brodmann areas 40, 44, and 45) in both sexes (females: 0.20 [<0.01] vs 0.12 [<0.01]; *P* < .001; males: 0.20 [<0.01] vs 0.12 [<0.01]; P < .001). Global efficiency of the entire cerebello-thalamo-cortical network was higher in survivors with executive dysfunction (mean difference, 0.0042; 95% CI, −0.0042 to 0.0040; *P* = .01). Within-module-degree *z* score of left DLPFC showed increased differences among survivors with impairment compared with survivors without impairment (Figure 1). In female survivors, global efficiency was inversely correlated with cognitive flexibility (Pearson *r* = −0.24; *P* = .02) and visual-motor processing (Pearson *r* = −0.27; *P* = .04). Individuals with executive dysfunction had increased global efficiency between smaller brain regions (Pearson *r* = −0.24; *P* = .01) compared with individuals without dysfunction. Associations between control language network and neurocognitive function were not significant.

### Effective Connectivity Analysis

**Figure 2.**
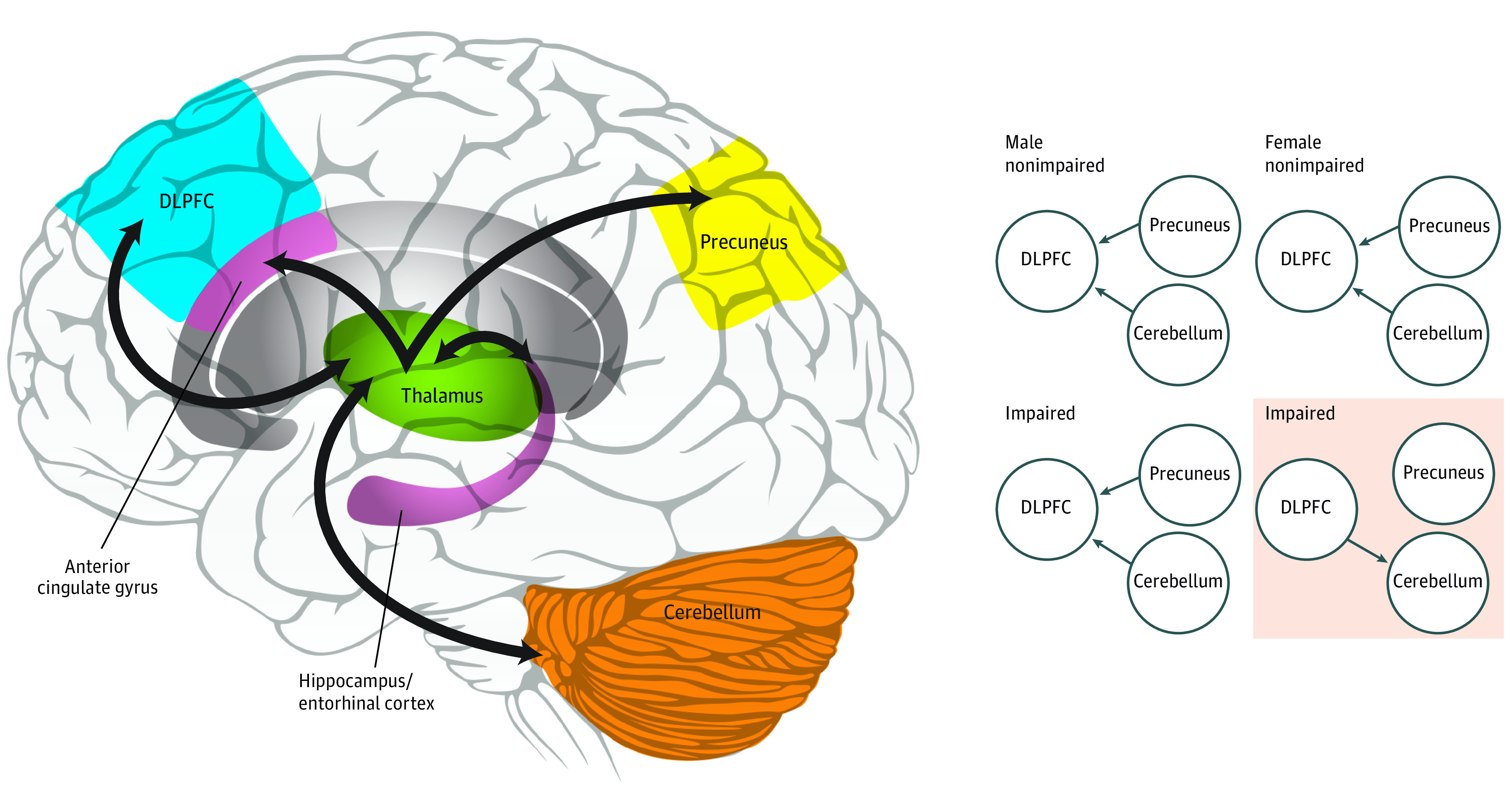
Bayesian Network Analysis of Cerebello-Thalamo-Cortical Network of Glucocorticoid–Sensitive Regions in Survivors Our results suggest that the cerebellum and precuneus are associated with DLPFC (dorsolateral prefrontal cortex) activity for all groups except female survivors with impairment (shaded box), who have differences in both directionality and connectivity. Gray brain regions indicate cingulum and ventricle and are used for neuroanatomical reference for the anterior cingulate gyrus, which is a substructure of the cingulum; bold arrows, neural connections between regions; nodes (ie, circles), brain regions; directed edges (ie, thin arrows), highest-probability connection and direction of interaction between regions; absence of edge (ie, absence of thin arrow), no probability of connectedness.

Using estimated effective connectivity network structure derived from bayesian network analysis, we found that DLPFC activity was conditionally dependent on activity in the cerebellum and precuneus in all cohorts without impairment (ie, there were no effects of sex). Estimated network structure was consistent in male survivors with impairment. However, in female survivors with impairment, there were differences in network connectivity and directionality (Figure 2). Specifically, cerebellar activity was conditioned by DLPFC activity, and there was no connectivity between DLPFC activity and precuneus activity. In ANOVA of connectivity weights derived from bayesian network analysis of the entire cohort, we identified a significant interaction association among the direction and strength of connectivity between the cerebellum and DLPFC, female sex, and executive dysfunction (*F* = 3.99; *P* = .02).

## Discussion

This cross-sectional study found decreased cerebellar volume in survivors of childhood ALL, who were treated with intravenous methotrexate, oral dexamethasone, and intrathecal glucocorticoids and methotrexate, compared with a control population. Decreased cerebellar volume was associated with poorer performance in tests of working memory, organization and planning, and motor and visual processing speed. Cerebello-thalamo-cortical network functional connectivity measures were associated with poorer neurocognitive outcomes. Moreover, effective connectivity in female survivors with impaired executive function was significantly different compared with female survivors without impairment and male survivors with or without impairment. Our results suggest that the cerebellum and precuneus are associated with DLPFC activity for all groups except female survivors with impairment, who have differences in both directionality and connectivity. Consistent with the graph theory analysis, this highlights a different mechanism of network dysfunction in female survivors with impairment. Additionally, we found that global efficiency (a measure of total network integration and information exchange) was increased in the cerebello-thalamo-cortical network compared with a control network with fewer glucocorticoid receptors. High efficiency indicates high network integration and capacity for parallel information processing. However, beyond an optimal range, increased efficiency suggests excessive long-range connections, which are metabolically costly.^44,45^ This study’s findings suggest that glucocorticoids and methotrexate may be associated with disruption of substrates of the cerebello-thalamo-cortical neural network and with neurocognitive impairments seen in survivors of childhood ALL.

In children, healthy development includes a period of overconnectivity followed by a period of synaptic pruning.^46^ As such, higher global efficiency can be associated with altered or poorly pruned structural networks.^47^ Higher global efficiency is also associated with greater metabolic cost and shows an inverted, U-shaped association with cognitive function (ie, too little and too much connectivity are associated with poor neurocognitive performance).^44,48^ In female survivors in our study, global efficiency was inversely correlated with cognitive flexibility and visual-motor processing speed. In comparison, a 2016 study^49^ of 99 healthy preadolescent children found that global efficiency was positively associated with performance on measures of visual-motor processing. Moreover, higher global functional connectivity has been found among patients with mild cognitive impairment.^50^ Our results suggest that treatment may be associated with disruption of optimal brain network organization and function and with impairment of executive function seen in survivors of ALL.

Bilateral cerebello-thalamo-cortical networks appeared disconnected at the lowest-density threshold for a subset of survivors and could not be validly compared between groups by hemisphere. The fragmentation was not significantly different in survivors with executive dysfunction compared with those without executive dysfunction, but fragmentation may affect other neurocognitive functions we did not assess. It is biologically implausible that any brain region would be completely disconnected from all other regions. Thus, our finding that several survivors had such low connectivity that a common minimum density could not be found supports our hypothesis that disruption of the cerebello-thalamo-cortical network may be associated with neurocognitive problems seen in survivors of ALL. This finding could also reflect graph-defining properties, such as size (ie, number of nodes) of the cerebello-thalamo-cortical network, which is known to affect connectome measurement.^30^ However, evidence for altered connectivity was further suggested by bayesian network analysis, which found that impairment in female survivors was associated with a lack of effective connectivity between precuneus and DLPFC.

We found similarities and differences in neurocognitive-associated network changes by sex. Significant associations were found between dominant hand motor processing speed and left cerebellar volume in male and female survivors. In male survivors, visual processing speed was associated with bilateral cerebellar volume. These findings agree with a 2014 study^51^ that demonstrated an association between the cerebellum and motor/visual processing performance. In our study, poor Rey-Osterrieth complex figure performance was associated with decreased cerebellar volume and increased dexamethasone exposure in female survivors but not in male survivors. This is noteworthy given the results of the bayesian analysis, which suggested that in female survivors, impairment was associated with altered effective connectivity among the DLPFC, precuneus, and cerebellum. Lobule IX and Crus I and II of the cerebellum have been shown to project to the default mode network and may be associated with this disruption.^23,52^ Unfortunately, the caudal vermis, which contains lobule IX, was not reliably captured in many of our study’s fMRIs; therefore, we were not able demonstrate this lobule’s effective connectivity to the precuneus to test this hypothesis.

Sex differences were seen in this study, and these differences in outcomes may be associated with postnatal testosterone surge seen in newborn boys.^53^ A 1996 animal study^54^ demonstrated that androgen receptor binding mediates the downregulation of glucocorticoid receptor expression in CA1 pyramidal cells of the hippocampus in rats. This may indicate that androgen exposure in young men is associated with decreased sensitivity to glucocorticoid receptor–mediated effects. Additionally, a 2017 in vitro study^55^ found that 17-β estradiol levels were associated with modulating the effects of oxidative stress and may have protective associations for pubertal girls. This would suggest that prepubertal girls and individuals with hypogonadism may have increased sensitivity to intrathecal triple therapy and dexamethasone. An association between decreased hormone receptor expression and decreased functional connectivity between DLPFC and precuneus has been seen in survivors of breast cancer.^21^ Unfortunately, we did not obtain sex hormone levels and were unable to fully test this hypothesis. Nonetheless, sex differences in our findings may indicate that boys and girls require different strategies to improve neurocognitive outcomes.

Clinical implications of this study may include the need for strategies to investigate the use of n-acetyl cysteine (n-AC), which may be able to rescue the glutathione antioxidant system in individuals receiving chemotherapy. Studies^56,57^ have shown that n-AC has a good safety profile and is tolerable at doses necessary for central nervous system penetrance. Additionally, N-methyl-D-aspartate (NMDA) receptor antagonist has been associated with reversing of cortisol-induced glutamatergic synaptic pruning and may be associated with the decreased oxidative injury associated with glucocorticoids.^58^ However, studies are needed to investigate if these neuroprotective agents can decrease oxidative injury without compromising the efficacy of cancer therapy. Transcranial electrical stimulation devices have been associated with altered activity of resting-state networks and regulation of NMDA receptor activity and may offer a potential therapy for survivors after completion of therapy.^59,60,61,62^ Additionally, Children’s Oncology Group long-term follow-up guidelines do not include dexamethasone or intrathecal hydrocortisone as potential risk factor for cognitive late effects.^63^ The results of our study suggest that this may need to be reconsidered.

### Limitations

This study has some limitations, including the lack of availability of neurocognitive testing in the control population. We did not have measures for biomarkers associated with oxidative injury before or during therapy or high-resolution MRI results that could help exclude pretreatment oxidative injury associated with disease or genetic predisposition. Additionally, we cannot exclude potential effects of cytarabine on brain volumes.^64^ Furthermore, we performed a subgroup analysis of survivors with or without impairment for the resting-state fMRI analysis to test our hypothesis that cerebello-thalamo-cortical network disruption was associated with executive dysfunction. This type of subgroup analysis can increase risk of false-positive errors if done incorrectly. However, our study was designed to accommodate this type of analysis, based on empirical evidence and defined a priori hypothesis. These factors may reduce the probability of a type I error. Additionally, this study is a single-institution, cross-sectional analysis and, as such, is not representative of every chemotherapy-only ALL regimen. Doses of methotrexate and dexamethasone in this study were consistent with the current standard of care. Future studies should investigate other glucocorticoids to determine whether other formulations produce similar effects in those populations. These studies should include measures of short- and long-term memory in addition to the measures of working memory included in this study.^13^ Neuroimaging studies should include resting-state fMRI with complete coverage of the cerebellum.

## Conclusions

These findings suggest that intrathecal glucocorticoids and methotrexate and oral dexamethasone may be associated with disrupted functional connectivity and poorer neurocognitive outcomes in female survivors of childhood ALL. Furthermore, the findings suggest that cerebellum activity is associated with neural activity of the DLPFC and disruption of the association between the two is associated with executive dysfunction.

## Notes

Supplement. **eAppendix.** Supplemental Methods
