# Supplementary material for: Connectivity of the Cerebello-Thalamo-Cortical Pathway in Survivors of Childhood Leukemia Treated With Chemotherapy Only
Source: JAMA Netw Open. 2020 Nov 20;3(11):e2025839. doi: 10.1001/jamanetworkopen.2020.25839 (PMC7679952; doi:10.1001/jamanetworkopen.2020.25839)
Supplement: Supplement. — eAppendix. Supplemental Methods [file jamanetwopen-e2025839-s001.pdf]

Supplemental Online Content

Phillips NS, Kesler SR, Scoggins MA, et al. Connectivity of the cerebello-thalamo-cortical pathway in survivors of childhood leukemia treated with chemotherapy only. *JAMA Netw Open*. 2020;3(11):e2025839. doi:10.1001/jamanetworkopen.2020.25839

eAppendix. Supplemental Methods

This supplemental material has been provided by the authors to give readers additional information about their work.

© 2020 Phillips NS et al. *JAMA Network Open.*

eAppendix. Supplemental Methods

Imaging

Processing included motion correction and averaging of 3-D T1-weighted images.[1] Removal of non-brain tissues was conducted using a hybrid watershed/surface deformation procedure.[2] Automated Talairach transformation and segmentation of the subcortical white matter and deep grey matter volumetric structures was then performed.[3,4] Maps were created using spatial intensity gradients across tissue classes. The maps are not reliant on absolute signal intensity and can detect submillimeter differences between groups because they are not restricted to the voxel resolution of the original data. These procedures have been validated against histological studies and manual measurements.[5-7] Freesurfer morphometric procedures have proven to have good test-retest reliability across different scanner manufacturers and various field strengths.[8,9]

Resting state fMRI was obtained during six minutes of eyes open rest on a 3T scanner (Siemens Trio or Skyra MR; Siemens, Malvern, PA) using a single-shot T2*-weighted EPI pulse sequence (TR = 2.06s, TE = 30ms, FOV = 192mm, matrix = 64x64, slice thickness = 5mm). Images were processed using Statistical Parametric Mapping v8 (SPM8) as previously described [10-13] including slice-time correction, realignment, normalization and smoothing (8mm full width half maximum). Functional volumes were further de-noised to reduce motion and signal related artifacts using a wavelet de-spiking method.[14]

Functional connectivity analysis

Functional connectivity processing, using the CONN Toolbox, included filtering data to the <0.1 Hz range of spontaneous activity[15] and correction of motion and physiologic/non-neuronal artifacts.[16] Edges were defined as correlation coefficients calculated between fMRI

© 2020 Phillips NS et al. *JAMA Network Open.*

regional mean time courses for each pair of regions in standard space. The resulting z-score connectivity matrices were thresholded to minimum connection density and then submitted to graph theoretical analysis to measure relevant connectome properties.[4]

We measured the within module degree z-score (WMZ) of each region to indicate how well-connected the node was within its functional network. WMZ is defined as $z_i = \frac{k_i\,(m_i) - \bar{k}(m_i)}{\sigma_{k(m_i)}}$ where $k_i\,(m_i)$ is the within-module degree (number of connections linking node i to other nodes in the same module m_i, $\bar{k}(m_i)$ is the mean within-module degree of nodes in the same module as node i, and $\sigma_{k(m_i)}$ is the standard deviation of $k_i\,(m_i)$ across all nodes in module m_i.[17,18] WMZ scores were calculated at minimum connection density for each network and compared between groups using two-tailed Wilcoxon rank sum tests (due to their non-normal distribution).

We calculated global efficiency, defined as the inverse of the average shortest path between nodes, of each subnetwork. Global efficiency is an indicator of the network's integration and capacity for parallel information processing.[19] We measured global efficiency at each of multiple densities from minimum connection density to the last density of small-world organization (i.e. valid biological network) to obtain an AUC.[20] The AUCs for each group were then compared between groups using second level analysis with permutation testing (two-tailed, 2000 iterations).

Effective connectivity analysis

To identify network structure, we performed a Bayesian network analysis. This technique can be used to determine the conditional dependencies between brain regions that characterize effective connectivity using functional MRI data.[21] The input variables were the regional time

© 2020 Phillips NS et al. *JAMA Network Open.*

courses extracted from a 50-element independent component analysis (ICA) from our three regions of interest. All three regions of interest, (cerebellum, precuneus, & DLPFC) were well identified by the ICA. We used the R package bnlearn with an underlying Tabu search algorithm for network structure identification which was verified by a hill climbing search algorithm to estimate the effective functional connectivity structure for the 4 subgroups (male impaired, male non-impaired, female impaired, and female non-impaired). Impaired was defined by z-score on any executive function test < 1.3. and the entire cohort of survivors combined. Differences in subgroup networks organization were determined by inspection since our model contained a manageable number of nodes. The data from all survivors were fit to the network estimated from the complete cohort. A 2-way ANOVA was then used to identify sex, impaired/non-impaired, or interaction effects.

© 2020 Phillips NS et al. *JAMA Network Open.*

1. Reuter M, Rosas HD, Fischl B. Highly accurate inverse consistent registration: a robust approach. *Neuroimage.* 2010;53(4):1181-1196.
2. Segonne F, Dale AM, Busa E, et al. A hybrid approach to the skull stripping problem in MRI. *Neuroimage.* 2004;22(3):1060-1075.
3. Fischl B, Salat DH, Busa E, et al. Whole brain segmentation: automated labeling of neuroanatomical structures in the human brain. *Neuron.* 2002;33(3):341-355.
4. Fischl B, van der Kouwe A, Destrieux C, et al. Automatically parcellating the human cerebral cortex. *Cereb Cortex.* 2004;14(1):11-22.
5. Kuperberg GR, Broome MR, McGuire PK, et al. Regionally localized thinning of the cerebral cortex in schizophrenia. *Arch Gen Psychiatry.* 2003;60(9):878-888.
6. Salat DH, Buckner RL, Snyder AZ, et al. Thinning of the cerebral cortex in aging. *Cereb Cortex.* 2004;14(7):721-730.
7. Rosas HD, Liu AK, Hersch S, et al. Regional and progressive thinning of the cortical ribbon in Huntington's disease. *Neurology.* 2002;58(5):695-701.
8. Han X, Jovicich J, Salat D, et al. Reliability of MRI-derived measurements of human cerebral cortical thickness: the effects of field strength, scanner upgrade and manufacturer. *Neuroimage.* 2006;32(1):180-194.
9. Reuter M, Schmansky NJ, Rosas HD, Fischl B. Within-subject template estimation for unbiased longitudinal image analysis. *Neuroimage.* 2012;61(4):1402-1418.
10. Kesler SR, Gugel M, Pritchard-Berman M, et al. Altered resting state functional connectivity in young survivors of acute lymphoblastic leukemia. *Pediatr Blood Cancer.* 2014;61(7):1295-1299.
11. Kesler SR, Blayney DW. Neurotoxic Effects of Anthracycline- vs Nonanthracycline-Based Chemotherapy on Cognition in Breast Cancer Survivors. *JAMA Oncol.* 2016;2(2):185-192.
12. Kesler SR, Wefel JS, Hosseini SM, Cheung M, Watson CL, Hoeft F. Default mode network connectivity distinguishes chemotherapy-treated breast cancer survivors from controls. *Proc Natl Acad Sci U S A.* 2013;110(28):11600-11605.
13. Bruno J, Hosseini SM, Kesler S. Altered resting state functional brain network topology in chemotherapy-treated breast cancer survivors. *Neurobiol Dis.* 2012;48(3):329-338.
14. Patel AX, Kundu P, Rubinov M, et al. A wavelet method for modeling and despiking motion artifacts from resting-state fMRI time series. *Neuroimage.* 2014;95:287-304.
15. Whitfield-Gabrieli S, Ford JM. Default mode network activity and connectivity in psychopathology. *Annu Rev Clin Psychol.* 2012;8:49-76.
16. Behzadi Y, Restom K, Liau J, Liu TT. A component based noise correction method (CompCor) for BOLD and perfusion based fMRI. *NeuroImage.* 2007;37(1):90-101.
17. Sporns O, Betzel RF. Modular Brain Networks. *Annu Rev Psychol.* 2016;67:613-640.
18. Newman ME. Modularity and community structure in networks. *Proceedings of the National Academy of Sciences of the United States of America.* 2006;103(23):8577-8582.
19. Latora V, Marchiori M. Efficient behavior of small-world networks. *Phys Rev Lett.* 2001;87(19):198701.
20. Bassett DS, Bullmore E, Verchinski BA, Mattay VS, Weinberger DR, Meyer-Lindenberg A. Hierarchical organization of human cortical networks in health and schizophrenia. *J Neurosci.* 2008;28(37):9239-9248.
21. Tzourio-Mazoyer N, Landeau B, Papathanassiou D, et al. Automated anatomical labeling of activations in SPM using a macroscopic anatomical parcellation of the MNI MRI single-subject brain. *Neuroimage.* 2002;15(1):273-289.

© 2020 Phillips NS et al. *JAMA Network Open.*
